# TET2 in epigenetic control of immune cells: Implications for inflammatory responses and age-related pathologies

**DOI:** 10.1016/j.jbc.2026.111267

**Published:** 2026-02-06

**Authors:** Tomasz Obrebski, Marta Maleszewska, Stanislaw Dunin-Horkawicz, Anna R. Malik

**Affiliations:** 1 Cellular Neurobiology Research Group, Institute of Developmental Biology and Biomedical Sciences, Faculty of Biology, University of Warsaw, Warsaw, Poland; 2 Department of Animal Physiology, Institute of Experimental Zoology, Faculty of Biology, University of Warsaw, Warsaw, Poland; 3 Department of Protein Evolution, Max Planck Institute for Biology Tübingen, Tübingen, Germany; 4 Institute of Evolutionary Biology, Faculty of Biology, Biological and Chemical Research Centre, University of Warsaw, Warsaw, Poland

**Keywords:** DNA methylation, cytokine expression, clonal hematopoiesis, neurodegeneration, atherosclerosis, ischemic stroke

## Abstract

Ten–eleven translocation 2 (TET2) is an epigenetic modifier whose canonical activity leads to the removal of cytosine methylation in the genome, which in essence results in the activation of gene expression. This function is particularly well described in the context of hematopoiesis and its alterations that lead to leukemia. However, in recent years, it has become evident that the noncanonical functions of TET2 also play a vital role in its activity. Rather than depending on its catalytic activity, these functions arise from TET2 interactions with other epigenetic modifiers. This review summarizes the structure, regulation, and functions of TET2 in immune cells. We describe how TET2 controls gene expression at both the DNA and RNA levels. In addition, we discuss the role of TET2 in hematopoietic stem cell fate and in clonal hematopoiesis of indeterminate potential. Finally, we highlight the impact of TET2 mutations on age-related inflammatory diseases, including cardiovascular and neurodegenerative disorders. Collectively, available evidence positions TET2 as a key integrator of epigenetic state and immune signaling, with context-dependent effects on inflammation and tissue homeostasis, and underscores the therapeutic potential of targeting TET2-dependent pathways in clonal hematopoiesis and inflammatory diseases.

Ten–eleven translocation 2 (TET2) is an epigenetic modifier that belongs to the TET family of three methylcytosine dioxygenases (Fig. 1), with specificity for DNA and RNA. The first protein was discovered in 2002 and named after the type of mutation present in a patient with leukemia, TET1 (1, 2). TET2 and TET3 were discovered later based on sequence homology. Knowledge about TET proteins' function and activity came in 2009 with Tahiliani's article (3), where they discovered that TET1 converts 5-methylcytosine (5mC) to 5-hydroxymethylcytosine in mammalian DNA. This activity is key for controlling DNA methylation, which is one of the epigenetic mechanisms regulating gene expression. Attachment of a methyl group to cytosine is catalyzed by DNA methyltransferases and can be actively removed by the mechanism involving TET1/2/3 or by passive dilution during replication. DNA methylation is recognized as a repression mark, directly blocking binding of transcription factors (TFs) to chromatin, and indirectly by making DNA less accessible. Thus, this canonical activity of TETs facilitates active gene transcription.

**Figure 1:**
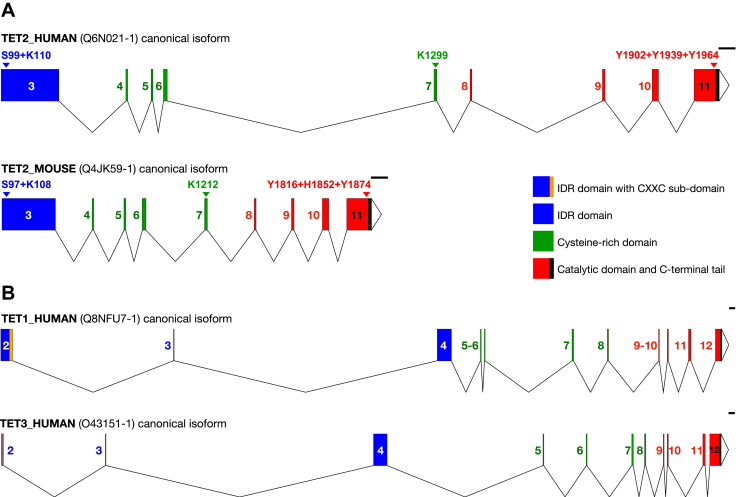
**Intron–exon structure of TET isoforms**. *A*, human *TET2* and mouse *Tet2*; (*B*) human *TET1* and human *TET3*. In all panels, *numbered boxes* represent exons and are color coded according to the domains they primarily encode: *blue*, N-terminal intrinsically disordered region (IDR); *green*, cysteine-rich domain; *red*, DSBH catalytic core; *orange*, CXXC motif; and *black*, C-terminal tail. 5′ and 3′ UTRs are omitted, and introns are shown to scale. Selected regulatory residues are indicated above the corresponding exons for human and mouse TET2, with numbering based on UniProt canonical isoform numbering. *Triangles* at the right end of each transcript mark the 3′ end of the last coding exon. *Black horizontal bars* denote a 1 kb scale. DSBH, double-stranded β-helix; TET, ten–eleven translocation.

TET1, mainly expressed in embryonic stem cells (ESCs), is responsible for their maintenance. Besides that, it plays a role in development, female germ cell formation, and spatial learning and memory (4). TET1 preferentially binds to cytosine–phosphate–guanine dinucleotides (CpGs) within the transcription start site and promoter flanking regions. Its occupancy positively correlates with CpG content; however, TET1 binds both active and repressed promoters, which might suggest its dual function in regulating gene expression (4). In mouse ESCs, about 50% of DNA methylation is removed by TET2, and the majority of TET2-binding sites is located within the gene body (5). TET2 is best known for its role in hematopoiesis and regulation of innate immunity (6). TET3, similarly to TET1, preferentially occupies gene promoters; however, it can bind both CpG and non-CpG sequences (7). TET3 regulates DNA methylation status during zygote formation, embryogenesis, axon regeneration, and synaptic transmission (6). In *Xenopus*, TET3 regulates the expression of key developmental factors during eye and neuronal development (7). TET1 and TET3, besides the catalytic core region, contain the CXXC domain, which is responsible for DNA binding. TET2 lacks this domain, likely because of chromosomal inversion, during which the TET2-binding domain transformed into an independent gene, *IDAX*, also known as *CXXC4* (6).

Canonical activity of TET2, which contributes to the removal of cytosine methylation in the genome (Fig. 2), in essence results in the activation of gene expression. This function is well described in particular in the context of hematopoiesis and its alterations leading to leukemia. However, in recent years, it has become evident that also the noncanonical functions of TET2, relying on its interactions with other epigenetic modifiers, play a vital role in its activities. This role appears particularly important in mature immune cells and usually leads to the opposite phenomenon, that is, gene expression silencing. In this review, we discuss the activities of TET2 with an emphasis on its noncanonical functions. We summarize the role of TET2 in regulating normal hematopoiesis, and we discuss in more depth its impact on the activities of immune cells, such as macrophages, neutrophils, and lymphocytes, in particular in modulating the expression of proinflammatory cytokines. These aspects of TET2 function are particularly relevant for TET2-related clonal hematopoiesis, which becomes more prevalent with age and is associated with worse outcomes of several diseases with an inflammatory component.

**Figure 2:**
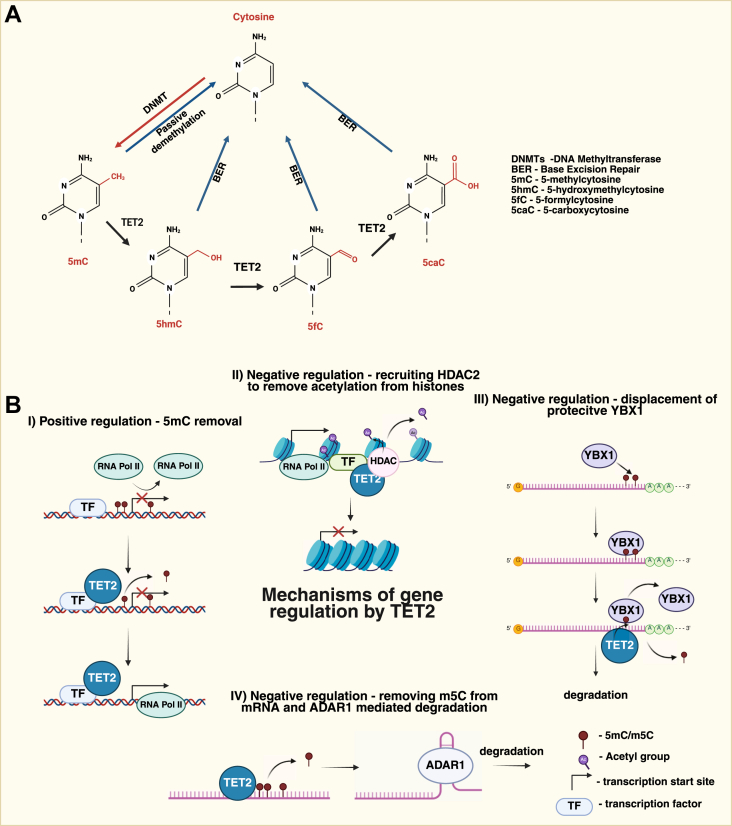
**The mechanisms of TET2-dependent gene expression control**. *A*, the mechanisms leading to 5mC removal from the genome. *B*, canonical and noncanonical mechanisms of TET2-dependent control of gene expression. (I) TET2, recruited by a transcription factor (TF), removes 5mC, facilitating active transcription. (II) TET2 acts as a scaffolding protein for histone deacetylase (HDAC), which removes acetyl groups from histone lysines, leading to chromatin condensation and gene silencing. (III) Y-box binding protein 1 (YBX1), acting as an m5C reader, binds to and protects the transcript from degradation. Oxidation of m5C by TET2 leads to YBX1 dissociation and transcript degradation. (IV) Removal of m5C enables formation of a hairpin-like structure, which is recognized by double-stranded RNA–specific adenosine deaminase (ADAR1), directing the transcript for degradation. 5mC, 5-methylcytosine; TET2, ten–eleven translocation 2.

## TET2 gene and protein

Human *TET2* is controlled by three closely spaced promoters (Pro1, Pro2, and Pro3) that generate alternative first exons and confer tissue- and stage-specific expression (8). Ensembl lists multiple transcripts, but at the protein level, UniProt consistently annotates three isoforms: the full-length canonical Q6N021-1 (2002 amino acids; matching ENST00000380013.9; 11 transcript exons; coding sequence in exons 3–11; associated with Pro2; Fig. 1) and two C-terminally truncated isoforms that lack the double-stranded β-helix (DSBH) catalytic domain, Q6N021-2 (1165 amino acids; matching ENST00000305737.6) and Q6N021-3 (1194 amino acids; matching ENST00000265149.9). The two shorter TET2 isoforms arise through alternative splicing coupled to premature 3′ termination (8, 9): the Pro1-derived transcript terminates at an intronic poly(A) site, producing the 1165-aa isoform (Q6N021-2) that retains sequence corresponding to exons 3 to 4 of the full-length canonical form but lacks the downstream region corresponding to exons 5 to 11; in contrast, the Pro3-derived isoform (Q6N021-3) terminates within a region corresponding to canonical exon 3 and lacks sequence corresponding to exons 4 to 11 of the full-length canonical form.

In mice, *Tet2* has a broadly similar exon–intron organization to humans but occupies a more compact locus owing to shorter introns (Fig. 1). Ensembl lists multiple transcripts, but at the protein level, UniProt curates a single canonical isoform, Q4JK59-1 (1912 amino acids; matching ENSMUST00000098603.8), and an additional full-length unreviewed product, A0A0G2JF55 (1920 amino acids; matching ENSMUST00000196398.5). However, in contrast to humans, where alternative promoter usage and splicing yield truncated isoforms, mouse TET2 is reported predominantly as full length, possibly from a promoter corresponding to human Pro2 (8). Beyond human and mouse, TET2 is conserved across vertebrates, with particularly strong conservation of the cysteine-rich (Cys-rich) and DSBH catalytic core domains (6).

In both human and mouse canonical isoforms, exon boundaries align approximately with the modular domain architecture: exon 3 contributes most of the N-terminal intrinsically disordered region (IDR), exons 4 to 7 largely encode the Cys-rich region, and exons 8 to 11 encode the DSBH catalytic core; exon 11 also encodes a short non–DSBH C-terminal tail. Both proteins lack the CXXC DNA-binding domain, which is instead encoded by the adjacent *CXXC4/IDAX* gene (10). TET2 domains are selectively lost in engineered and naturally truncated forms and harbor several important regulatory sites (see the “Regulation of TET2 activity” section and Table 1 for details).

**Table 1:** Post-translational modifications of TET2

Residue in human	Residue in mouse	Modification	Modifying enzyme(s)	Functional consequences	References
S99	S97	Phosphorylation	Promoted by AMPK (kinase); removed by PP2A (phosphatase—validated in human)	TET2 protein stabilization; increase in 5hmC levels	(11, 12, 58)
K110/K111 (validated with MS)	K108 (not validated)/I109 (cannot be acetylated)	Acetylation	Promoted by p300 (acetyltransferase); removed by HDAC1/HDAC2 (deacetylases)	TET2 protein stabilization; increase in 5hmC levels; increased DNMT1 binding and chromatin association (enhanced by p300/HDAC inhibition; reduced in 2KR mutant)	(13)
K1299	K1212	Monoubiquitination	Promoted by CRL4-VprBP (E3 ubiquitin ligase); removed by USP15 (deubiquitinase)	Promotes TET2 chromatin association (reversed by USP15)	(14, 15)
Y1902	Y1816 (not validated)	Phosphorylation	Promoted by FGFR3Δ7–9 (splice variant; kinase); phosphatase unknown	Increased ubiquitination and proteasome-mediated degradation of TET2	(18)
Y1939/Y1964	H1852 (cannot be phosphorylated)/Y1874 (not validated)	Phosphorylation	Promoted by JAK2 (kinase); phosphatase unknown	TET2 protein activation; increase in 5hmC levels	(16)

Briefly, within the N-terminal IDR, a protein–protein interaction platform, phosphorylation at S99 (S97 in mouse) has been linked to protein stabilization and phosphorylation-dependent interactions (11, 12). Acetylation at K110/K111 has also been described, primarily associated with increased stability and global 5-hydroxymethylcytosine (5hmC) levels (13). The aligned mouse site is K108, but its functional role has not been experimentally validated. In the Cys-rich region, a conserved monoubiquitination at K1299 promotes chromatin–DNA association (14, 15). The aligned mouse residue is K1212. In the human C-terminal tail downstream of the DSBH domain, Janus kinase 2 (JAK2) phosphorylates Y1939 and Y1964 (16). The aligned mouse residues are H1852 and Y1874; the former is histidine and thus not a JAK2 substrate, whereas the latter lacks experimental validation. Within the DSBH catalytic core, Y1902 (aligning to mouse Y1816) contributes to the active-site scaffold (17) and has been reported as phosphorylated in cancer (18).

Human TET2 is also regulated at the transcriptional level, and its catalytic core can be lost physiologically through expression of two naturally truncated isoforms (see above) that retain the N-terminal IDR and Cys-rich region but lack the DSBH; these isoforms have been proposed to act in a dominant-negative manner by competing with the full-length protein (19). An analogous loss of catalytic activity has been modeled in mouse by engineered deletion of *Tet2* exons 8 to 10 (Fig. 1) (20). Further studies have tested loss of TET2 activity using point mutations in key catalytic residues in exon 9 (21). In contrast, *Tet2* KO alleles commonly target exon 3, which contains the start codon (22, 23). Finally, targeting exon 11 enables testing the contribution of the C-terminal region *in vivo* (24).

## The mechanisms of TET2-dependent regulation of gene expression

In this section, we present the mechanism by which TET2, directly or indirectly, regulates gene expression in immune-related processes. Some of the mechanisms will be further described in more detail in the section dedicated to cytokine expression (see “TET2-dependent regulation of cytokine expression and secretion” section). Worth pointing out is the dual character of TET2 activity toward DNA—canonical, based on its catalytic activity, and noncanonical, relying rather on TET2 as a scaffolding protein for other chromatin remodelers (Fig. 2). Of note, as TET2 lacks a DNA recognition domain, its recruitment to specific genomic loci relies on interactions with chromatin-associated proteins (10). Strikingly, the spectrum of TET2-binding proteins is very broad. The DSBH domain located at the C-terminal part of TET proteins is the binding site for most of the known partners, whereas only a few interact with the N-terminal regions (9). All TET proteins interact with O-GlcNAc transferase (OGT), CTCF, and base excision repair DNA glycosylase (9). TET2 interactors primarily belong to three groups: TFs, histone modifiers, and signaling regulators. TET2 is known to interact with TFs, such as the components of the NF-κB pathway (during inflammation, see later), or early B-cell factor 1, a key TF in the regulatory network in the pre–pro-B cell stage (25, 26, 27). Importantly, TET2 interacts with chromatin modifiers like histone deacetylases (HDACs, see later), INO80, SMARCC2, and SMARCB (9, 26). In addition, there are several hardly classifiable TET2 interactors, such as PROSER1, which stabilizes TET2–OGT interactions and links them with the UTX complex, or CXXC5, helping to regulate gene expression (28, 29). These are examples of known TET2 interactors, which are extensively described in other reviews (9, 27, 29, 30).

Thus, targeting TET2 to specific genomic loci depends on its interactions with TFs (Fig. 2*B*). Several TFs have been shown to recruit TET2 to DNA during its canonical activity, including GATA3, WT1 (31, 32), and the NF-κB subunit p65 (RelA) (25). In the case of the noncanonical activity of TET2, the role of NF-κB inhibitor zeta (IκBζ) in TET2 recruitment to DNA was documented (26). Strikingly, the components of the NF-κB pathway (RelA and IκBζ) appear to be involved in TET2 recruitment in both functional modes. The NF-κB pathway is activated during immune responses after the recognition of pathogen-associated molecular patterns (PAMPs, such as lipopolysaccharide [LPS]) or damage-associated molecular patterns (DAMPs, such as mitochondrial DNA [mtDNA], ATP, and others) by specialized receptors, including Toll-like receptors (TLRs). Upon activation, TLRs initiate downstream signaling pathways (such as the NF-κB pathway) and, in turn, cytokine production and secretion. TLRs can be located at the cell surface (*e*.*g*., TLR1, TLR2, TLR4, and TLR5) or in endosomes (*e*.*g*., TLR3, TLR7, TLR8, and TLR9) (33, 34).

Canonical activity of TET2, followed by the steps catalyzed by other enzymes, ultimately leads to the removal of 5mC (Fig. 2, *A* and *B* I). In the enzymatic reaction performed by TET2, 5mC is oxidized to 5hmC, which can be further transformed to 5-formylcytosine and 5-carboxylcytosine, and finally replaced with unmodified cytosine *via* the base excision repair mechanism (35, 36) (Fig. 2A). The resultant replacement of 5mC with cytosine facilitates gene transcription. Regulation of gene expression by the canonical function of TET2 begins early in life, during embryonic development (5, 37). This mechanism is widely used by many cell types as a positive regulator of transcription. The canonical function of TET2 was shown to be crucial for proper hematopoiesis (see the “Role of TET2 in development and hematopoiesis” section). In macrophages, TET2 oxidizes 5mC during activation of innate immunity (25). Detailed mechanisms and biological contexts are described in the following sections.

Noncanonical activity of TET2, in turn, is activity independent and relies on the interactions with other chromatin remodelers. Here, TET2 acts as a scaffolding protein, facilitating the enzymatic activity of its partners (Fig. 2*B* II). Such cooperation is best documented for the role of TET2 in facilitating the removal of histone acetylation. This process, mediated by HDACs, is a key mechanism of gene silencing. For example, TET2, in cooperation with HDAC2 and IκBζ, facilitates transcription repression of TLR-activated signaling pathway targets in dendritic cells and macrophages. During the early stages of LPS stimulation, IκBζ binds to promoters, increasing the expression of proinflammatory genes. At the late stages of LPS stimulation of murine macrophages, IκBζ recruits TET2 to the *Il6* promoter. TET2, in turn, interacts with HDAC2, facilitating the removal of the acetyl group from histones and silencing of transcription (26). Similar noncanonical TET2/HDAC-dependent mechanisms of gene silencing were also described for *Il1b* (38, 39) (see also “TET2-dependent regulation of cytokine expression and secretion” section) and *Mif* (40), genes encoding proinflammatory cytokines. Silencing of *Mif* transcription depends on histone deacetylation by HDAC1, which is recruited to the *Mif* gene promoter by TET2 interacting with a TF EGR1 (early growth response protein 1). TET2 mutant without catalytic activity was capable of blocking the expression of *Mif* (40), substantiating the notion that this mechanism is activity independent.

In recent years, the number of studies examining the role of TET2 in post-transcriptional regulation of gene expression at the RNA level has been growing. Methylation of mRNA, known as “m5C” in this case, is recognized and bound by its readers, protecting mRNA from decay and extending its lifetime. This post-transcriptional regulatory mechanism leads to prolonged expression without active transcription. TET2 activity, leading to the removal of the m5C mark, was shown to promote mRNA degradation (Fig. 2*B* III). Here, Y-box binding protein 1 (YBX1) binds m5C in mRNA, protecting the transcript from degradation. TET2-mediated removal of m5C, depleting YBX1 from mRNA, leads to its degradation, as shown for the *Tspan13* transcript (41). The protective effect of m5C also applies to the *Klf4* and *Rock1* transcripts (42). In the second mechanism of mRNA stability control by TET2, removal of the m5C mark influences RNA secondary structure, which enables binding of a double-stranded RNA-specific deaminase, and ultimately leads to transcript degradation (43) (Fig. 2*B* IV). This mechanism has been described for *Socs3* mRNA, which encodes a key suppressor of the JAK–signal transducer and activator of transcription signaling pathway activated by cytokines, interferons, and growth factors. In detail, TET2 controls *Socs3* expression at the level of mRNA by modifying its 3′UTR, which triggers the formation of a hairpin-like structure bound by adenosine deaminase acting on RNA 1 (ADAR1). ADAR1 activity leads to destabilization of the transcript and reduced translation (43).

In summary, TET2 actions can lead both to gene expression activation and to repression, depending—among others—on the availability of the interacting partners. Thus, the consequences of TET2 deficiency are highly context-, time-, and cell type–dependent and may differ significantly between the studies, which will be further discussed in sections “TET2-dependent gene regulation: Conclusions from transcriptome analyses” and “TET2-dependent regulation of cytokine expression and secretion.”

## Regulation of TET2 activity

**Figure 3:**
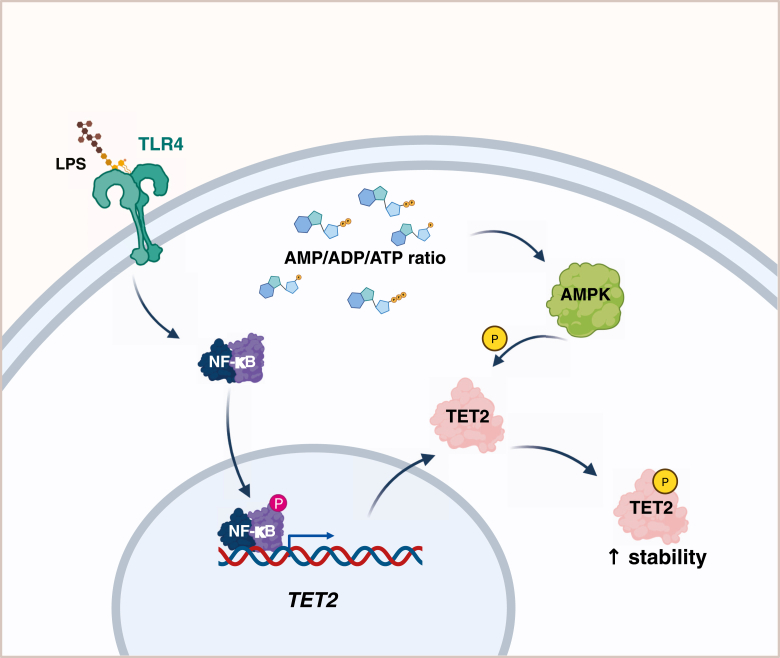
**Main mechanisms regulating TET2**. Cellular levels of TET2 are regulated by two major pathways: Toll-like receptor (TLR) and AMP-activated kinase (AMPK) signaling. Activation of TLRs by their ligands, such as LPS, triggers NF-κB signaling, leading to enhanced transcription of the *TET2* gene. AMPK, activated when energy levels are low, phosphorylates TET2, which results in its stabilization. LPS, lipopolysaccharide; TET2, ten–eleven translocation 2.

TET2 is subject to dynamic regulation of its cellular levels and enzymatic activity. The mechanisms underlying this control include transcriptional regulation in response to extracellular stimuli as well as post-translational modifications (Fig. 3).

### Regulation of TET2 mRNA levels

The mechanisms regulating TET2 expression are only partially understood. Studies in murine cells showed that the levels of *Tet2* mRNA increase upon proinflammatory stimulation, but this phenomenon is not fully consistent between stimulants and cell types. Proinflammatory stimulant LPS is thus far the best described trigger for *Tet2* induction (Fig. 3), but its (patho)physiological relevance is limited to bacterial infections. LPS induces *Tet2* expression in the microglial BV2 cell line and in primary murine and rat microglia (44) as well as in peritoneal macrophages (PMs), bone marrow (BM)–derived macrophages, and macrophage-like cell lines RAW264.7 (45) and J774.1 (46). This induction is mediated *via* activation of the TLR4 receptor and the NF-κB pathway (44, 45). At the same time, *Tet1* and *Tet3* were not affected by LPS treatment. Other ligands of the TLRs, such as lipoteichoic acid (ligand for TLR2) as well as fibronectin (that potentiates TLR4 signaling, most likely *via* an integrin β1–dependent mechanism (47, 48)) also triggered *Tet2* upregulation (44). Of note, to the best of our knowledge, there are no published data showing regulation of TET2 levels in response to inflammatory stimulation by cytokines, such as interleukin 1β (IL-1β) or tumor necrosis factor alpha (TNF-α). So, it is unclear whether *Tet2* gene expression can be dynamically regulated in immune cells during inflammatory responses in contexts other than bacterial infection.

There are several reports showing *Tet2* expression changes in response to other, noninflammatory factors. BV2 cells treated with α-synuclein aggregates and amyloid-β (Aβ) oligomers for 6 h showed an increase of *Tet2* expression similar to that observed after LPS (44). Furthermore, 17β-estradiol was shown to upregulate *TET2* mRNA levels in the human breast cancer cell line MCF7 (49). In detail, *TET2*, but not *TET1* or *TET3*, is a 17β-estradiol–responsive gene. Various populations of immune cells, in particular neutrophils (50), are regulated by estrogen, so it is tempting to speculate that this mechanism might have relevance for modulating TET2 levels in these cells.

Finally, several miRNAs were shown to regulate *TET2* transcript levels. Already in 2013, Cheng *et al*. (51) performed an unbiased screen to identify miRNA-mediated regulation of *TET2* 3′ UTR. Over 30 miRNAs were shown to inhibit *TET2* expression and cellular 5hmC, including miR-125b, miR-29b, miR-29c, miR-101, and miR-7. Later studies led to further discoveries of *TET2*-regulating miRNAs. For example, depleting Let-7 microRNA prevented the LPS-dependent increase of *Tet2* expression in murine macrophages (52).

### Post-translational regulation of TET2

Post-translational modifications, such as phosphorylation, acetylation, ubiquitylation, and potentially also glycosylation (53), are emerging as key factors regulating TET2 activity (Table 1). These post-translational modifications often affect TET2 activity indirectly by influencing protein degradation. It has been reported that TET2 can be degraded by three different mechanisms: caspase-dependent degradation (10), calpain-mediated proteolysis (54), and proteasomal degradation (13, 55). Both calpains and caspases are cysteine proteases involved, among other processes, in protein turnover (56, 57). While calpains are calcium-dependent proteases with broad substrate specificity, caspases show a strict requirement for aspartate residue before the cleavage site. The degradation of TET2 by calpain and proteasomes was shown to be subject to regulation by post-translational modifications.

Phosphorylation of TET2 at S99 (in human, which corresponds to S97 in mouse) has been characterized as an important regulatory modification introduced by the AMP-activated kinase (AMPK, Fig. 3) (11, 12). The phosphorylation at this residue in human cells was shown to be removed by protein phosphatase 2A (58). In consequence, the AMPK pathway, which is the central energy sensor of cells, primarily activated by high AMP/ATP or ADP/ATP ratios, emerges as the key pathway regulating TET2 levels and, in consequence, the levels of 5hmC in the genome (11, 58, 59). This mechanism was mostly studied in the context of the links between cancer and metabolic disturbances, such as diabetes and obesity. In detail, AMPK-mediated phosphorylation of TET2 at S99 stabilizes the protein (11). Increased glucose levels impede this modification, leading to destabilization of TET2, followed by dysregulation of 5hmC levels. Several cell lines (PBMC, HUVEC, and TF-1) exhibited significantly lower levels of 5hmC when subjected to high as opposed to normal glucose, which coincided with a decrease of TET2 levels in these cells. Conversely, activation of AMPK results in stabilizing TET2 (11). The mechanism behind AMPK-dependent stabilization of TET2 is likely mediated by the protection from calpain-mediated proteolytic degradation, assured by S99 phosphorylation. Another study focusing on the myogenic differentiation pointed to an alternative or perhaps complementary mechanism mediated by the 14-3-3 proteins (12). This study demonstrated that binding of TET2 to 14-3-3β is S97 phosphorylation dependent and that increasing amounts of 14-3-3β resulted in increased levels of TET2. The stabilizing effect of S99 phosphorylation and the 14-3-3 protein interaction with phosphorylated TET2 were further confirmed in another study (58). How these mechanisms may relate to the activities of TET2 in immune cells has not been studied thus far. However, given the important function of the AMPK pathway in these cells (60, 61), the functional implications of this mechanism are highly likely.

Tyrosine phosphorylation of TET2 has also been reported (16, 18). First, FGFR3Δ7–9, a splicing mutant of fibroblast growth factor receptor 3, relevant for hepatocellular carcinoma, was shown to phosphorylate TET2 at Y1902 in human cells. This phosphorylation promoted ubiquitination and proteasomal degradation of TET2 (18). Second, a regulatory axis linking factors regulating hematopoiesis and TET2 activity was described in hematopoietic stem/progenitor cells and erythroid progenitors (16). Unlike other mechanisms discussed thus far, this one appears to directly control the enzymatic activity and not the protein stability of TET2. In detail, TET2 is phosphorylated by JAK2 in response to hematopoietic cytokines, such as erythropoietin, stem cell factor, and FLT3 ligand. This phosphorylation occurs at Y1939 and Y1964, located within the catalytic core of human TET2, and results in TET2 activation and subsequently in a global increase in 5hmC levels in the genome. Removing these two tyrosine residues from TET2 interrupted hematopoietic colony formation and terminal differentiation of erythroid cells. Conversely, the JAK2-activating mutation V617F was associated with increased hydroxymethylation and decreased genome cytosine methylation in primary myeloproliferative neoplasm samples and transgenic mouse models. Interestingly, the two identified sites of tyrosine phosphorylation within TET2 are absent in TET1 and TET3, suggesting nonoverlapping functions for TET2.

Another post-translational modification of TET2 is acetylation introduced by p300 acetyltransferase (13) and removed by HDAC1/2. The acetylated site was identified as K110/K111 residues (in human protein). Acetylation at K110/K111 increased DNA methyltransferase 1 binding and chromatin association. Insulin treatment induces TET2 acetylation and promotes its activity, as presumed from increased global 5hmC levels without changes in TET2 protein levels. In line with the proposed mechanism, knocking down either *HDAC1* or *HDAC2* also increased 5hmC levels. In longer time scales, acetylation also stabilized TET2 by inhibiting ubiquitination and proteasomal degradation. Of note, in this study, the sirtuin (SIRT) inhibitor nicotinamide did not affect TET2 acetylation in the A2780 ovarian cancer cell line, whereas SIRT1 was reported to be a TET2 deacetylase in the SH-SY5Y neuroblastoma cell line (55). Here, the activity of SIRT1, modulated with the use of its agonist SRT1720 or inhibitor EX-527, was negatively correlated with the protein levels of TET2. In agreement with the previous report, deacetylation of TET2 promoted TET2 degradation *via* the ubiquitin–proteasome pathway.

Finally, monoubiquitination (which should not be confused with polyubiquitination, often promoting proteasomal degradation) was also documented to regulate TET2. TET2 is monoubiquitylated at K1299 (in humans, corresponding to K1212 in mice), which promotes its binding to DNA (15). Monoubiquitination of TET2 is promoted by VprBP/DCAF1 (15) and removed by USP15 (14).

In summary, TET2 levels and activity are regulated in response to external cues (*e*.*g*., the presence of pathogens, cytokines) and by the metabolic status of the cell. This dynamic regulation adjusts TET2 activity to the current needs during the processes that strongly depend on TET2, such as development and hematopoiesis.

## Role of TET2 in development and hematopoiesis

The role of TET2 in embryonal development and hematopoiesis, particularly in humans, has been intensively studied. TET2 expression is low in human ESC lines and increases during hematopoietic differentiation. In mouse embryos, loss of individual *Tet* genes or *Tet1/Tet2* together does not disrupt hematopoiesis (62, 63, 64). However, complete or endothelial-specific loss of all three TET enzymes after gastrulation reduces hematopoietic stem and progenitor cells (HSPCs) and causes midgestation lethality. This results from impaired HSPC specification from endothelial cells (ECs) in the yolk sac and aorta–gonad–mesonephros region. *Tet* deficiency leads to hypermethylation and downregulation of NF-κB1 and key hematopoietic TFs (GATA1/2, RUNX1, and GFI1B), highlighting their role in epigenetic control of the EC-to-hematopoietic stem cell (HSC) transition (65). *TET2* silencing in human ESCs skewed their differentiation into neuroectoderm and decreased the development of the mesoderm germ layer through a downregulation of NANOG but without altering their pluripotency (66). Consequently, hematopoiesis was greatly impaired, as demonstrated by a decrease in both the number and the cloning capacities of hematopoietic progenitors (66). However, when *TET2* was knocked down in later stages of development in hematopoietic cells, hematopoietic development was promoted.

TET2 depletion affects both murine and human adult hematopoiesis by enhancing the self-renewing potential of HSCs and favoring monocytic differentiation at the expense of granulopoiesis (66, 67, 68). Moreover, mice conditionally expressing miR-22, which targets *Tet2* in HSCs, displayed increased HSC self-renewal with defective hematopoietic differentiation and developed myelodysplastic syndrome (MDS) similar to TET2-deficient mice, further highlighting its role in hematopoiesis (69). *In vitro*, TET2 deficiency delayed HSC differentiation and skewed development toward the monocyte–macrophage lineage (20). In competitive transplantation assays, TET2-deficient HSCs were capable of multilineage reconstitution and possessed a competitive advantage over WT HSCs (20). However, *Tet2*-null HSCs unexpectedly exhaust at the same rate as WT HSCs in serial transplantation assays despite an initial increase in self-renewal, suggesting that the effect of TET2 depletion manifests more profound myeloid lineage skewing in committed hematopoietic progenitor cells rather than long-term HSCs (70).

TET2 deficiency is also associated with disordered lymphopoiesis and erythropoiesis in both human and mouse models (21, 71). For example, knockdown of *TET2* in human cord blood CD34^+^ cells skews progenitor differentiation toward the granulomonocytic lineage at the expense of lymphoid and erythroid lineages (68) TET2 deficiency leads initially to stem cell factor–dependent hyperproliferation and impaired differentiation of human colony-forming unit–erythroid cells (72). Furthermore, TET2 is also involved in JAK2-mediated erythropoietin signaling. Cytokine stimulation leads to JAK2 activation, which in turn phosphorylates TET2. Phosphorylated TET2 interacts with the erythroid TF, KLF1, thereby promoting transcription of erythroid genes and differentiation of erythroid progenitors (16). *In vivo*, *Tet2* catalytic mutants, which preserve the noncanonical TET2 activity, *versus Tet2*^*−/−*^ HSPCs that lose both TET2 functions, exhibit distinct gene expression profiles. While *Tet2* mutant mice predominantly developed myeloid malignancies resembling human MDSs, only one-third of *Tet2* KO mice developed MDS-like phenotypes. In a small subset of *Tet2* KO mice (<5%), the predominant alteration was a moderate increase in CD3e^+^ T cells. Notably, over one-fourth of *Tet2* KO mice exhibited aberrant lymphopoiesis, primarily involving B-cell abnormalities, which were more frequent than myeloid or erythroid dysplasia. HSPCs from *Tet2* KO mice exhibited distinct gene expression profiles, including downregulation of *Gata2*. Overexpression of *Gata2* in *Tet2* KO BM cells ameliorated disease phenotypes (21).

TET2 deficiency was also recently shown to impair NK cell maturation. While its deletion in hematopoietic cells or immature NK cells does not affect early NK development, it blocks terminal maturation marked by CD11b, CD43, and KLRG1. In the liver, *Tet2* loss hinders NK maturation, increases the proportion of type 1 innate lymphoid cells, and decreases conventional NK cells. Hematopoietic *Tet2* deletion also reduces perforin and eomesodermin/Tbr2 TF levels in NK cells (73). TET2 also affects human neutrophil development and function. TET2 loss (premature stop in exon 11) in human HSCs produces a distinct neutrophil heterogeneity in BM and peripheral tissues by increasing the repopulating capacity of neutrophil progenitors and giving rise to low-granule neutrophils. Human neutrophils that inherited *TET2* mutations mount exacerbated inflammatory responses and have more condensed chromatin, which correlates with compact neutrophil extracellular trap production (74). Altogether, TET2 is a master regulator of hematopoiesis balance since embryonic development through the lifespan, up to late stages of life.

TET2 has a critical role in regulating the expansion and function of HSCs, presumably by controlling 5hmC levels at genes important for the self-renewal, proliferation, and differentiation of HSCs (66, 71). Loss of TET2 disrupts this regulation, leading to aberrant chromatin accessibility and increased expression of self-renewal and proliferation-associated genes. For example, TET2 deficiency increases H3K79 dimethylation and expression of *Mpl*, which encodes the thrombopoietin receptor. Inhibition of *Mpl* expression or the signaling downstream of the thrombopoietin receptor is sufficient to reduce the competitive advantage of murine and human TET2–deficient HSPCs (75).

TET2 (and TET3), in cooperation with OGT, regulate chromatin states by influencing both protein GlcNAcylation and histone H3K4 methylation through the COMPASS (Complex Proteins Associated with Set1) complex. Host cell factor 1—a structural component of the COMPASS complex that plays a role as a regulatory unit—is a direct GlcNAcylation substrate of OGT. This post-translational modification of host cell factor 1 is essential for maintaining COMPASS integrity and activity. TET2/3 and OGT colocalize at active promoters, where they promote recruitment of the H3K4 methyltransferase SETD1A—another member of the COMPASS complex— thereby supporting transcriptional activation. Loss of TET2/3 or OGT leads to reduced H3K4me3 and transcriptional downregulation, as observed in TET2-deficient mouse BM, which shows globally decreased GlcNAcylation and H3K4me3 at genes critical for hematopoiesis (76).

Moreover, the pre-existing epigenetic landscape of cells influences how they respond to TET2 loss, explaining the variable phenotypic outcomes of identical mutations (77, 78). Recently, single-cell analyses for RNA-Seq, DNA methylation, and assay for transposase-accessible chromatin sequencing have shown that TET2 loss drives methylation of accessible TF-binding sites, such as those of erythroid TFs. This leads to attenuated binding of erythroid TFs, including KLF1 and TAL1, to CpG-rich erythroid motifs, which then induces a block of erythroid differentiation and skewed differentiation to myelomonocytic lineage in TET2-deficient HSCs (77). Together, these findings highlight the complex interplay between TET2-mediated epigenetic regulation and HSC fate, with implications for understanding its phenotypic differences.

Overall, the role of TET2 in development and hematopoiesis remains the best studied aspect of TET2 biology. The importance of this role is highlighted by the alterations of hematopoiesis resulting from TET2 dysfunction or deficiency.

## Alterations of hematopoiesis triggered by TET2 dysfunction

**Figure 4:**
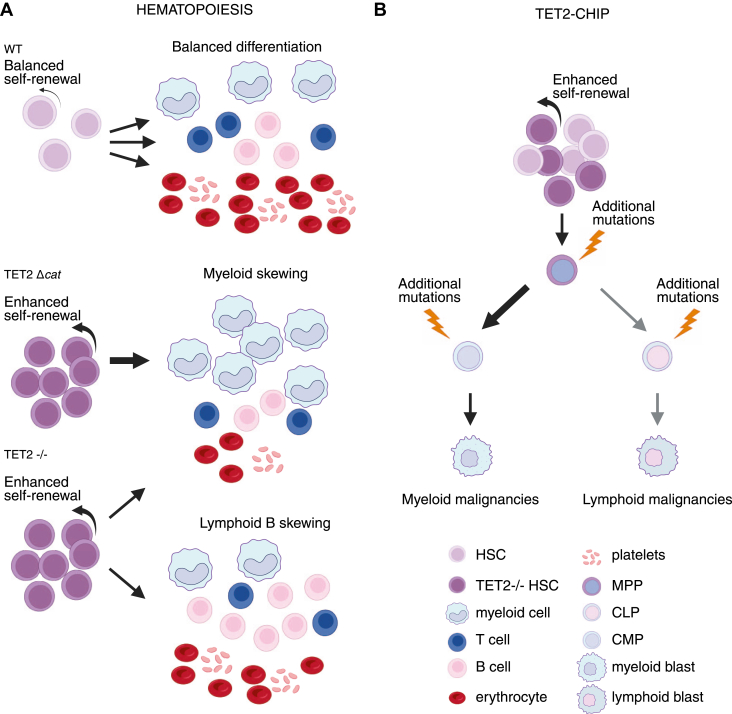
**Effects of TET2 loss on hematopoietic balance and disease progression**. Effects of TET2 loss on hematopoietic stem cell fate in mouse models. WT hematopoietic stem cells (HSCs) maintain balanced self-renewal and differentiation into myeloid, lymphoid, and erythroid lineages. Alterations in TET2 enhance HSC self-renewal and alter lineage output: TET2 catalytic mutant (TET2 Δcat) exhibits skewed differentiation characterized by a predominant myeloid bias with reduced lymphoid and erythroid contribution, whereas loss of TET2 (TET2^−/−^) results in expansion of either the myeloid or the lymphoid compartment, predominantly affecting B cells, with rare involvement of T lymphocytes. *B*, TET2-deficient HSCs gain a competitive advantage and undergo clonal expansion consistent with clonal hematopoiesis of indeterminate potential (TET2-CHIP). Acquisition of additional mutations in TET2-deficient progenitors promotes progression to hematologic malignancies, most commonly myeloid and, less frequently, lymphoid. CLP, common lymphoid progenitor; CMP, common myeloid progenitor; MPP, multipotent progenitor; TET2, ten–eleven translocation 2.

*TET2* mutations frequently occur in various types of hematological conditions, ranging from clonal hematopoiesis of indeterminate potential (CHIP) to leukemias (Fig. 4). CHIP refers to the clonal expansion of HSCs carrying cancer-associated mutations without developing malignancy. The variant allele frequency of mutated cells in CHIP is equal to or greater than 2% of blood cells. Somatic mutation rate increases with age; it is estimated that 10% to 20% of individuals above 70 years can carry a CHIP-related mutation (79). Mutations in many genes can cause CHIP; however, up to 87% of all CHIP cases are linked to mutations in the genes encoding epigenetic modifiers, including TET2, which is linked to 33% of CHIP cases (79, 80, 81, 82). The mechanism of developing CHIP is gene specific, and not all of them have been described yet; however, they share common patterns. In brief, mutations present in HSCs result in increased proliferation and self-renewal capacity, causing over-representation of mutated effector cells like monocytes, leukocytes, and neutrophils in the blood (81). Clonal expansion of TET2-deficient cells is promoted by inflammatory conditions (83, 84). TET2-deficient murine BM progenitors exhibit a proliferative advantage under TNF-α and interferon gamma (IFN-γ) stress compared with their WT counterparts (83). In line with these notions, in mice treated with IL-1, *Tet2*^+/−^ HSPCs and mature blood cells develop TET2-CHIP (85). Conversely, antibiotic treatment to suppress infection-driven inflammation as well as pharmacological inhibition of TNF-α signaling attenuated growth of TET2-deficient hematological malignancies *in vivo* (84).

CHIP increases the relative risk of cancer (81, 86) and the risk of developing blood malignancies like acute myeloid leukemia (AML) or MDS. In other words, CHIP can evolve into blood malignancy in some patients. Over longer time scales, *Tet2*-null mice develop myeloid and also, less frequently, T- and B-cell malignancies (22, 23, 87, 88). This transition from CHIP to malignancy likely requires accumulating additional malignancy-driving mutations in TET2-deficient cells. Recent data demonstrate that TET2 loss leads to hypermutagenicity in HSPCs. *Tet2*^−/−^ mice spontaneously developed lethal hematological malignancies that accumulated numerous mutations, including *Apc*, *Nf1*, *Flt3*, *Cbl*, *Notch1*, and *Mll2*, which are recurrently deleted/mutated in human hematological malignancies. Murine Lin^−^c^−^Kit^+^ HSPCs showed higher mutation frequencies in *Tet2*^−/−^ cells, particularly at genomic sites that gained 5hmC, where TET2 normally binds. Furthermore, *TET2*-mutated myeloid malignancy patients have significantly more mutational events than patients with WT *TET2* (87). Mechanistic insights into the underlying cause of this phenomenon are provided by Xie *et al*., who demonstrated that DNA damage arising in TET2-deficient HSPCs leads to activation of the cyclic guanosine monophosphate-adenosine monophosphate synthase (cGAS)–stimulator of interferon genes (STING) pathway, which in turn promotes the enhanced self-renewal and expansion of mutated clones. Both pharmacological inhibition and genetic deletion of STING suppress *Tet2* mutation–induced aberrant hematopoiesis. Moreover, in patient-derived xenograft models, STING inhibition specifically attenuates the proliferation of leukemia cells from *TET2*-mutated individuals (89).

Implications of TET2 deficiency in hematopoietic cells for hematopoietic malignancies are best described for AML. Here, loss of TET2 enhances leukemic stem cell (LSC) homing to the BM niche, thereby promoting LSC self-renewal and disease persistence (41). TET2 deficiency upregulates tetraspanin-13 (*TSPAN13*), which amplifies CXCR4/CXCL12 signaling and facilitates LSC trafficking to the BM niche. Elevated expression and activation of CXCR4/CXCL12 signaling ultimately enhances LSC homing and leukemic maintenance (41). In addition, TET2 cooperates with the myeloid TF CEBPA, which is frequently biallelically mutated in AML. In this context, TET2 is recruited to the *GATA2* enhancer to maintain GATA2 expression; consequently, loss of TET2 leads to increased *GATA2* promoter methylation, altering GATA2 levels and providing a selective advantage for leukemic cells. Importantly, demethylating therapy can restore *GATA2* expression and delay disease progression in *CEBPA-TET2* comutated AML (90). TET2 deficiency also synergizes with mutations affecting the PU.1 coding gene, where deletion of an upstream regulatory element in TET2-deficient mice results in highly penetrant, transplantable AML. Leukemic stem and progenitor cells show hypermethylation at putative PU.1-binding sites, fail to activate myeloid enhancers, and are hallmarked by a signature of genes with impaired expression shared with human AML (91). Furthermore, TET2 loss leads to reduced H2AK119ub levels, promoting open chromatin and increased transcription in stem cells. *TET2*-mutant human leukemia becomes dependent on this gene activation pathway, as depletion of MBD6 selectively impairs proliferation of these leukemic cells and partially rescues hematopoietic defects (92). In chronic myelomonocytic leukemia, *TET2* mutations frequently co-occur with overexpression of the histone demethylase KDM6B, jointly driving transcriptional dysregulation of proinflammatory and genome stability pathways (93).

Collectively, these findings highlight the central role of TET2 in safeguarding hematopoietic gene regulation and demonstrate how its loss creates vulnerabilities to malignant transformation. In addition to its implications for hematological malignancies, TET2-CHIP is associated with age-related diseases, which will be discussed in the “TET2 deficiency in the diseases associated with inflammation” section. Clear links between TET2 dysfunction and human disease have inspired transcriptomic studies focused on TET2-dependent gene regulation, in particular in myeloid cells.

## TET2-dependent gene regulation: Conclusions from transcriptome analyses

A classical approach to investigate the regulatory role of TET2 in gene expression is to combine TET2 depletion with a stimulation driving cell activation. That way, the consequences of TET2 removal for the transcriptome of activated cells can be investigated, allowing for the discovery of TET2-regulated genes. Several studies have been performed using this approach across various cell types, including microglia and macrophages, and usually applying LPS as a stimulant. Strikingly, published data are often contradictory (Table 2). Whether this is caused by the differences in the cell type, stimulant, time frame, or the exact approach used to deplete TET2 remains unclear. For example, comparing the effects of the LPS treatment in TET2-deficient and control BV2 microglial cells, the researchers found enrichment for inflammatory and cell cycle–related genes as well as genes related to intracellular signaling and TFs among TET2-regulated targets (44). Of note, cytokine genes were not among the hits reported in this study. LPS treatment did not change global 5hmC levels, despite the increase in *Tet2* expression in the control cells upon this treatment. Furthermore, no significant changes were detected in the levels of 5mC in the promoters of selected target genes. At the same time, TET2 binding increased substantially at target genes upon LPS treatment. Thus, TET2-dependent gene expression changes in this model result from its noncanonical actions other than catalytic activity.

**Table 2:** Summary of the studies investigating TET2-dependent gene expression control

Cell type/genotype/stimulation	Method/Gene Expression Omnibus accession number/reference	Selected genes upregulated in TET2-deficient cells compared with nonmodified cells	Selected genes downregulated in TET2-deficient cells compared with nonmodified cells
		GO terms for genes upregulated in TET2-deficient cells compared with nonmodified cells	GO terms for genes downregulated in TET2-deficient cells compared with nonmodified cells
BV2/*Tet2* KD/LPS 1 μg/ml, 3 h	RNA-Seq/GSE105155/(44)	*Cdca2*, *Mybl2*, *Atad2*, *Ccne2*, *Asf1b*, *Mcm2*, *Aurkb*, *Fam64a*, *Brca2*, *Bub1b* (reported directly in the publication)	*Tor3a*, *Mnda*, *Anxa1*, *Isg20*, *Socs3*, *Socs2*, *Trim26*, *Mndal*, *Slfn8*, *Isg15*, *Ifi204*, *Irf1*, *Stat1*, *Stat2* (reported directly in the publication)
		Cell cycle, cell cycle processes, DNA replication, DNA metabolic process, mitotic cell cycle (reported directly in the publication)	Innate immune response, immune response, immune system processes, defense response, response to IFN-β (reported directly in the publication)
MDM/*TET2* KD/no stimulation	RNA-Seq/GSE206030/(110)	*TRIL*, *IDO1*, *NEURL3*, *IL27*, *HES4*, *FBXO39*, *CCL8*, *CXCL11*, *APOBEC3A*, *APOBEC3B-AS1*, *APOBEC3A_B*, *ISG20*, *CXCL10*, *DEFB1*, *FAM71A*, *NUPR1*, *IFITM1*, *RSAD2*, *GBP1P1*, *ETV7*, *SOCS1*, *TNFSF10*, *KIR2DL4*, *BEX2*, *CH25H*, *MIR3945HG*, *ZBP1*, *IFIT2*, *CCNA1*, *C1R* (top 30 genes reported in GSE206030 dataset consistently for both ASO)	*DEPTOR*, *C20orf197*, *GPR34*, *ADORA3*, *ADGRA3*, *PALD1*, *LGI2*, *CCDC152*, *REPS2*, *MARC1*, *CENPK*, *FGD5*, *LOC101929331*, *PID1*, *MRO*, *FAM111B*, *TMIGD3*, *POLQ*, *NTSR1*, *SELENOP*, *DTNA*, *CDCA7*, *ADAM12*, *TICRR*, *CCDC152*, *HGF*, *LOC145694*, *LRRC20*, *KDELC1*, *RNF125* (top 30 genes reported in GSE206030 dataset consistently for both ASO)
		Cytokine signaling in the immune system, interleukin cosignaling, IFN-α/β signaling, signaling by interleukins, chemokine receptor binding chemokines, IFN signaling, class I rhodopsin receptors, IL-4 and IL-13 signaling, peptide ligand binding receptors, GPCR ligand binding (reported directly in the publication)	Cell cycle, cell cycle mitotic, cell cycle checkpoints, mitotic G1 phase and G1–S transition, G0 and early G1, polo like kinase mediated events, resolution of sister chromatid, mitotic prometaphase, G1–S-specific transcription, G2–M checkpoint (reported directly in the publication)
BMDM/*Tet2* KO/no stimulation	RNA-Seq/GSE104828/(127)	*Itln1*, *Ms4a4c*, *Mmp9*, *Fgl2*, *Cd300e*, *Oasl1*, *Ndrg1*, *Cd5l*, *Klra2*, *Tagap*, *Nfkbiz*, *Slamf9*, *Itga9*, *Zbp1*, *Tnf*, *Isg15*, *Slco3a1*, *C3*, *Dst*, *Hmga1-rs1*, *Batf*, *Glrx*, *Sema4c*, *Tmem176b*, *Tnip3*, *Ehd1*, *Ccr5*, *Ms4a6b*, *Sipa1l1*, *Aif1* (top 30 genes reported in GSE104828 dataset)	*Asb10*, *Itgax*, *Fosb*, *Slco2b1*, *Tmem154*, *Entpd1*, *Fblim1*, *Enpp1*, *Atp6v0d2*, *Fabp4*, *Hal*, *Abca9*, *Tmem37*, *Bhlhe40*, *Hpse*, *Hpgd*, *Fam46c*, *Fcgr3*, *Klf9*, *Ccrl2*, *Cgnl1*, *Mmp12*, *Abcd2*, *Nuak1*, *Itgb3*, *Mical1*, *Rpl29*, *Gas6*, *Ephx1*, *Timp2* (top 30 genes reported in GSE104828 dataset)
		Positive regulation of leukocyte migration, positive regulation of cellular component movement, leukocyte activation, leukocyte differentiation, positive regulation of cytokine production, regulation of bone remodeling, regulation of osteoclast/osteoblast differentiation, regulation of bone mineralization, cytokine response, macrophage differentiation (reported directly in the publication)	Cellular response to organic substance, cellular response to chemical stimulus, response to organic substance, chemical homeostasis, cellular response to endogenous stimulus, cellular response to hormone stimulus, regulation of multicellular organismal development, response to endogenous stimulus (reported directly in the publication)
PMs/*Tet2* KO (Δex 8–10)/LPS 10 ng/ml + 2 ng/ml IFN-g, 10 h	Microarray/GSE81398/(38)	*Ccl3*, *Ccl4*, *Cxcl1*, *Cxcl13*, *Cxcl2*, *Cxcl3*, *Cxcl5*, *C4b*, *Dcn*, *Il12b*, *Il1a*, *Il1b*, *Il6*, *Osm*, *Il1rl2*, *Tlr2*, *Clec7a*, *Clec4n*, *C5ar1*, *Cd14*, *Itgb8*, *Itga4*, *Itgam*, *Itga6*, *Itgav*, *Lbp*, *Trafk1*, *Irak3* (top 28 genes reported directly in GSE81398 dataset)	*Scgb1a1*, *Ccl8*, *Hamp*, *Snora36b*, *Ifi30*, *Btg3*, *Snora81*, *Thbs4*, *Plau*, *Snora30*, *Slfn4*, *Ifi213*, *Aldh1b1*, *Daglb*, *Sp140*, *Rprl1*, *Clec4a3*, *Snora70*, *Fabp4*, *Snord43*, *Otulinl*, *Snora73a*, *Snora75*, *Snora73b*, *Syngr1*, *Dpy19l3*, *Sass6*, *Eps8*, *Qpct*, *Mfge8* (top 30 genes reported directly in GSE81398 dataset)
		Regulation of biological process, gland development, cell proliferation involved in kidney development, regulation of programmed cell death (GO analysis performed for this review based on the genes reported in GSE91398 dataset)	(GO analysis impossible—too few genes reported in GSE91398 dataset)
BMDC/*Tet2* KO (Δex 3)/LPS 100 ng/ml, 8 h	RNA-Seq/GSE69256/(26)	*Il10*, *U90926*, *Il6*, *Cxcl10*, *Il12b*, *Ptgs2*, *Rsad2*, *Il27*, *Il1b*, *Tnfsf15*, *Il12a*, *Cxcl1*, *Socs3*, *Il1a*, *Ifit1*, *Cmpk2*, *Ccl4*, *Oasl1*, *Cd40*, *Ifit2*, *Gbp5*, *Ifi205*, *Cxcl11*, *Tnf*, *Csf3*, *Il23a*, *Ch25h*, *Tgtp1*, *Cd69*, *Dusp14* (top 30 genes reported in GSE69256 dataset)	*Cd300lb*, *Lpar5*, *D930048N14Rik*, *Npas4*, *Lpin1*, *Cbx8*, *Mafb*, *Dnajc28*, *Pald1*, *Dnajb13*, *Oxld1*, *St6gal1*, *Susd3*, *Clec1a*, *Ypel2*, *Cnnm2*, *Cnr2*, *Dscc1*, *Slc26a11*, *Rassf7*, *Ung*, *Eps8*, *Sox4*, *Pdxk*, *Sh2d1b1*, *Map2k6*, *Kbtbd3*, *Kifc3*, *Irak1bp1*, *Sgsh* (top 30 genes reported in GSE69256 dataset)
		Cellular response to interferon-beta, response to interferon-beta, response to virus, cellular response to LPS, defense response to virus (GO analysis performed for this review based on the genes reported in GSE69256 dataset)	DNA repair, DNA metabolic process, cellular response to DNA damage stimulus, DNA replication, cellular response to stress (GO analysis performed for this review based on the genes reported in GSE69256 dataset)
BMDM/*Tet2* KO(Δex 3)/LDL 200 mg/dl, 24 h	RNA-Seq/(118)	*Il6*, *Cxcl3*, *Cxcl2*, *Il1b*, *Pf4*, *Accod1*, *Saa3*, *Ier3*, *Ifitm1*, *Serpinb2*, *Cd38*, *Fcrls*, *Trem1*, *Il1r1*, *Gpr84*, *Flrt3*, *Dmpk*, *Sema3c*, *Hspa1b*, *Gpr162*, *Marcksl1*, *Nptx1*, *Tgfb1*, *Dmwd* (reported directly in the publication)	No information available
		Cytokine–cytokine receptor interaction, focal adhesion, ECM receptor interaction, NOD-like receptor signaling pathway, cell adhesion molecules (top 5 KEGG terms from those reported directly in the publication)	Lysosome, drug metabolism cytochrome P450, selenoamino acid metabolism, regulation of autophagy, oxidative phosphorylation (top 5 KEGG terms from those reported directly in the publication)

GO terms presented in the table come from the listed publications; otherwise, they were generated based on publications data, using the PANTHER Classification System and a statistical over-representation test.

The GO term analysis performed for this review was based on statistically significantly upregulated/downregulated genes (log_2_[fold change] >1.5 and log_2_[fold change] <-0.5) reported in the datasets in the Gene Expression Omnibus repository. Analysis was performed with the PANTHER Classification System (PANTHER Over-representation Test) for the GO biological process.

ASO, antisense oligonucleotide; BMDC, bone marrow–derived dendritic cell; BMDM, bone marrow–derived macrophage; GO, Gene Ontology; KD, knockdown; MDM, monocyte-derived macrophage.

In contrast, another study using a microarray approach showed a prominent increase in the expression of multiple proinflammatory cytokines in TET2-deficient PMs in comparison to WT cells (38). Here, a combination of LPS and IFN-γ was used to stimulate the cells, and the strategy for TET2 depletion relied on removing exons 8 to 10. The expression of proinflammatory cytokines, including *Il1b*, *Il6*, *Cxcl3*, as well as PAMP receptors (TLR2) and signaling molecules (*Cd14*, *Itgam*, *Traf1*, and *Irak3*), was increased in TET2-deficient cells. The levels of 5hmC were not tested in this study. However, increased histone H3 acetylation at the *Il1b* promoter seen in TET2-deficient cells upon LPS/IFN-γ treatment points again to a mechanism independent of TET2 catalytic activity.

Key results of the RNA-Seq studies focusing on TET2-regulated genes are summarized in Table 2. In essence, they point to the involvement of TET2 in the control of inflammation-related genes, although the underlying mechanisms remain unclear at the moment. In addition to these reports, there are a number of studies focusing on particular cytokines as potential TET2 targets.

## TET2-dependent regulation of cytokine expression and secretion

Although not all transcriptome analyses point to genes encoding cytokines as the targets for TET2-dependent regulation, expression of several cytokines has been extensively studied in this context using targeted approaches. The role of TET2 in cytokine expression and release has attained substantial attention because of the relevance of TET2 for immune system functions and its links to diseases with an inflammatory component. Data exist that point to the regulatory role of TET2 in the expression and/or release of IL-1β, IL-6, and TNF-α. Of note, reported results are not always consistent and might, as in the case of analyses summarized in the previous section, depend on the cell type, the stimulant, and the time frame under investigation. How these expression levels correspond to the amount of released cytokines is also not always clear.

IL-1β is a potent proinflammatory cytokine released primarily by the cells of the monocytic lineage (monocytes, macrophages, and dendritic cells) and microglia *via* a noncanonical mechanism that does not rely on its transport through the ER–Golgi pathway (94, 95). Instead, IL-1β release depends on the inflammasome and passive release through the pores in the cell membrane created by gasdermin D or through ABC membrane transporters. In detail, IL-1β is present in the cytosol as pro-IL-1β, which requires proteolytic processing by activated inflammasome component NLRP3 (NOD-, LRR-, and pyrin domain–containing protein 3) to be secreted from the cell. Thus, the levels of the components of these pathways directly impact the secretion of IL-1β.

TET2 takes part in the regulation of *Il1b* transcription, although the direction of the regulation remains inconsistent between the studies. In BV2 cells treated with LPS, loss of TET2 exhibited time-dependent effects on *Il1b* expression with no differences observed after 3 h of treatment and a reduced expression of *Il1b* in TET2-deficient cells at 6 and 24 h post-LPS treatment (44). By contrast, in cultured PMs treated with a combination of LPS and IFN-γ, TET2 deficiency led to an increase in *Il1b* mRNA levels (38) as well as in the protein levels of IL-1β and its precursor form. As a result, TET2-deficient cells released more IL-1β, which was NLRP3 inflammasome dependent. HDAC inhibition increased *Il1b* expression in WT cells and abolished the differences between WT and *Tet2*^−/−^ cells, suggesting that acetylation rather than DNA methylation is responsible for the increased *Il1b* expression in TET2-deficient cells. In line with this notion, histone H3 acetylation at the *Il1b* promoter was greater in TET2-deficient macrophages. Finally, in an *ex vivo* stimulation assay, a significant induction of IL1-β was only observed in the whole blood of the TET2-deficient and not WT mice. However, the direct comparison of the LPS-challenged WT and *Tet2*-KO groups did not show significant differences (96). *In vivo*, macrophages isolated from mice after TET2-deficient BM transplantation displayed elevated *Il1b* transcript expression (97).

Processing of pro-IL-1β is also indirectly regulated by TET2. TET2 deficiency increased NLRP3 inflammasome expression in LPS/IFN-γ-treated macrophages (98). In detail, the inactive, ubiquitinated form of NLRP3 is phosphorylated by JNK in response to PAMP/DAMP, which in turn leads to deubiquitination by BRCC3 and formation of the active inflammasome and processing of pro-IL-1β. *Tet2* mutant macrophages exhibited hyperphosphorylation of JNK and increased IL-1β secretion, which was mediated by the impact of TET2 on the methylation status of the *Dusp10* promoter, whose gene product encodes a negative regulator of JNK (98).

IL-6 is a cytokine that can have both proinflammatory and anti-inflammatory properties depending on the context. IL-6 is secreted by activated immune cells and stromal cells, including T cells, monocytes/macrophages, ECs, fibroblasts, astrocytes, and hepatocytes. Interestingly, TET2 affects the responsiveness of cells to IL-6. In TET2-deficient macrophages, induction of the CSF1R in response to IL-6 was stronger than in WT cells (99).

Nevertheless, most studies focus on the role of TET2 in the control of IL-6 release. Similar to IL-1β, its expression was increased in TET2-deficient macrophages treated with LPS/IFN-γ (44) or LPS (52). Also, in an *ex vivo* setup, TET2-deficient mice showed increased serum levels of IL-6 upon LPS challenge of whole blood samples (96), and macrophages isolated from TET2-deficient mice displayed elevated *Il6* transcript expression (97). Again, there is some discrepancy between different studies. In the work by Carrillo-Jimenez *et al*. (44), TET2-deficient microglia released less IL-6 upon LPS stimulation than WT cells. Furthermore, in human macrophages differentiated under hypoxic conditions and treated with LPS, pharmacological inhibition of TET2 activity resulted in high DNA methylation levels and decreased expression of IL-6 and other inflammatory proteins (100).

**Figure 5:**
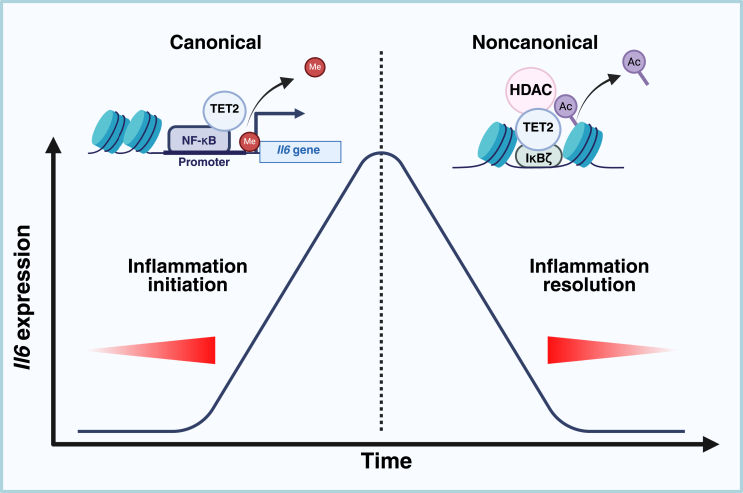
**Biphasic regulation of IL-6 expression by TET2**. Both the canonical and noncanonical activities of TET2 regulate IL-6 expression. The canonical activity of TET2, leading to the removal of cytosine methylation, facilitates *IL6* transcription in the acute phase of inflammation. By contrast, in the phase of inflammation resolution, noncanonical function of TET2 contributes to histone deacetylation by HDAC and IL-6 expression silencing. In both phases, TET2 is recruited to the DNA by various components of the NF-κB pathway. HDAC, histone deacetylase; IL-6, interleukin 6; TET2, ten–eleven translocation 2.

In fact, it is plausible that TET2 involvement in the regulation of IL-6 is biphasic, which can explain the contradictory results reported in different studies. Specifically, both the canonical and noncanonical activities of TET2 emerge as important regulators of IL-6 release (Fig. 5). While the canonical activity of TET2 facilitates IL-6 production in the acute phase of inflammation, the noncanonical function is important for its silencing in the phase of inflammation resolution. Potentially, this biphasic involvement of TET2 in IL-6 regulation may exemplify a mechanism of broader significance, relevant for the regulation of other proinflammatory mediators. The role of the noncanonical activity of TET2 in the regulation of IL-6 production was elucidated by Zhang *et al*. (26). TET2 was needed for active repression of *Il6* transcription during inflammation resolution, and *Il6* mRNA levels remained increased for a longer time in the case of TET2 depletion in several cell types: bone marrow–derived dendritic cells, BMDMs, and PMs. Thus, although TET2 deficiency did not affect the initial increase of *Il6* mRNA levels during LPS stimulation, it resulted in a prolonged *Il6* mRNA increase in TET2-deficient cells at the stage when its levels had already decreased in control cells. Also *in vivo*, after systemic challenge with LPS, conditional TET2-deficient mice produced more IL-6, especially at the late phase. Mechanistically, during the late phase of LPS treatment, TET2 binds to the *Il6* promoter, which is mediated by IκBζ. In turn, TET2 recruits HDAC2, which removes acetylation marks and silences transcription. In another study, however, histone H3 acetylation at the promoter of *Il6* was not changed in TET2-deficient microglial cells treated with LPS, compared with WT cells (44).

Another proinflammatory cytokine studied in the context of TET2-dependent regulation is TNF-α, released mainly by activated macrophages but also NK cells and T lymphocytes. Following 4-h *ex vivo* stimulation of the whole blood samples with LPS, TNF-α levels were higher in the samples from *Tet2*^−/−^ mice compared with WT mice (96). Furthermore, TET2 was shown to remove methylation from the *Tnf* promoter in a p65-dependent manner, facilitating its expression and secretion from macrophages infected with *Mycobacterium tuberculosis* (25). Another study, however, showed no alterations in *Tnf* expression levels in TET2-deficient LPS-stimulated murine BM-derived dendritic cells, BMDMs, and PMs, or *in vivo* in the blood serum of mice injected with LPS (26).

In summary, several important proinflammatory cytokines have been shown to be regulated by TET2. Moreover, as detailed in the “Alterations of hematopoiesis triggered by TET2 dysfunction" section above, TET2 plays a critical role in the control of hematopoiesis. Thus, it is not surprising that the impact of TET2 on immune cell activities has been gaining increasing attention in recent years.

## TET2 in the regulation of immune cell activities

TET2 has been extensively studied in the context of immune response, including both innate and adaptive responses. In particular, its activities emerge as an important factor regulating the differentiation and function of lymphocytes and myeloid cells.

### Lymphocytes

TET2 may regulate DNA 5hmC and, in consequence, gene expression, CD4^+^ and CD8^+^ T- and B-cell maturation and activation in many pathologies, including allergic response, autoimmune disease, and cancer (101, 102, 103, 104, 105). In T lymphocytes, TET2 modulates lineage-specific cytokine gene expression through the regulation of 5hmC at key regulatory elements, thereby influencing the differentiation and effector functions of Th1 and Th17 cells. Loss of TET2 reduces cytokine production, including IFN-γ and IL-17, by decreasing 5hmC levels and impairing the recruitment of TFs and coactivators such as p300 (102). In CD8^+^ T cells, TET2 deficiency enhances effector responses, as evidenced by increased IFN-γ and degranulation marker CD107a following lymphocytic choriomeningitis virus peptide stimulation (106). Moreover, TET2 regulates the epigenetic remodeling of the IFN-γ locus during CD8^+^ T-cell priming, promoting stable cytokine expression in response to proinflammatory stimulation with IL-12 (107). In B lymphocytes, TET2 activation enhanced IL-10 expression in regulatory B cells, contributing to immunosuppression and liver cancer progression, whereas adoptive transfer of TET2-deficient B cells suppressed tumor advancement (108). Together, these findings highlight the context-dependent role of TET2 in shaping both proinflammatory and regulatory immune responses across T- and B-cell subsets, impacting the pathogenesis of autoimmune diseases, allergic inflammation, and tumor immunity.

### Monocytes and macrophages

The activities of monocytes and macrophages that arise from monocytes are particularly affected by TET2 dysfunction. Already the precursor cells, monocytes, show an enhanced proinflammatory profile upon TET2 deficiency: the proportion of Ly6C^hi^ (proinflammatory) monocytes was higher in *Tet2*^−/−^ mice, and these cells responded to LPS stimulation with increased release of TNF-α and IL-6 (96). Also the mobilization of inflammatory monocytes to the blood is increased in *Tet2*^−/−^ mice (96). This may result from increased expression of the C-C chemokine receptor type 2 (CCR2), which is required for leukocytes, and especially Ly6C^hi^ monocytes, to leave the BM and enter the blood. The population of Ly6C^hi^ monocytes in the BM of *Tet2*^−/−^ mice showed higher levels of CCR2 than in the WT mice.

The primary function of TET2, in addition to driving macrophage differentiation, is to regulate the innate immune response. At the early stage of inflammation, the canonical function of TET2 activates expression of proinflammatory cytokines, whereas at the late stages, its noncanonical function helps to resolve it (Fig. 5). Stimulation with LPS or PAMPs promotes expression and secretion of proinflammatory cytokines in a TET2-dependent manner (25, 26). However, TET2-deprived macrophages are characterized by prolonged inflammation and cytokine secretion (26). Numerous data indicate elevated serum levels of IL-6, IL-1β, or MIF in TET2-CH individuals (40, 109). Usually, they are expressed and secreted in response to pathogen infections or tissue damage. One of the leading sources of those proinflammatory cytokines are monocytes and macrophages. TET2-depleted macrophages actively transcribe interferon-stimulated genes, leading to secretion of IFN-α and activation of bystander cells, even in the absence of PAMPs or DAMPs. Expression of TFAM, a mitochondrial TF, depends on TET2 interactions with RBPJ and ZNF143 at its regulatory elements. TET2 alteration leads to reduced expression of TFAM, affecting mitochondrial functioning and resulting in mtDNA leakage. mtDNA present in the cytosol is sensed by cGAS, which implements the interferon-stimulated gene program through activation of the STING signaling pathway (110). As a result, activities of *TET2*-mutated macrophages can lead to constant inflammation. Interestingly, TET2 also emerges as an important player during M2 activation of macrophages during allergic rhinitis. TET2 depletion leads to increased allergic inflammation and M2 polarization of macrophages characterized by increased expression of arginase 1 in BMDMs (42).

### Microglia

Microglia are resident immune cells of the central nervous system. They secrete cytokines and perform phagocytosis in response to injuries and the presence of DAMPs/PAMPs. Various extracellular debris and protein aggregates, including Alzheimer's disease (AD) hallmark Aβ peptides, are phagocytosed by microglia, which leads to their activation toward proinflammatory phenotypes. AD model animals transplanted with *Tet2*^−/−^ BM show a significantly higher number of peripheral macrophages compared with WT animals. Furthermore, those cells differentiate into microglia-like cells, expressing Tmem119, a marker of microglia (111). Human *TET2*^−/−^ induced pluripotent stem cells differentiated into microglia-like cells exhibit enhanced Aβ phagocytosis and proinflammatory cytokine production compared with WT cells (111). Thus, TET2 in microglia acts as a negative regulator of inflammation, and its loss may be beneficial in AD, which will be discussed in more detail in the “TET2 deficiency in the diseases associated with inflammation” section.

Microglia mediate neuropathic pain in response to nerve injuries. They secrete proinflammatory cytokines, which lead to abnormal neuronal activation in the dorsal horns of the spinal cord, causing neuropathic pain. Nuclear factor of activated T-cell (NFAT) is a TF that mediates TLR4-mediated microglia activation in response to nerve injuries, which is necessary in neuropathic pain development. TET2 acts as a positive regulator of inflammation and demethylates the *Nfat* promoter. *Tet2* KO animals show drastically reduced *Nfat* expression, whereas *Nfat* KO reduces microglia activation and abolishes neuropathic pain (112).

### Neutrophils

TET2 has an important role in regulating neutrophil development and function, as concluded from several studies in mouse models and in patients with clonal hematopoiesis. Overall, TET2 dysfunction enhances the generation of neutrophils, but these cells tend to show compromised function and an immature phenotype.

To investigate the impact of *TET2* mutations on hematopoiesis, Encabo *et al*. (74) utilized a model in which *TET2* loss-of-function mutations (premature stop in exon 11) were introduced to human HSPCs (CD34^+^CD38^-^), which were then transplanted into humanized immunodeficient mice. CyTOF mass cytometry analysis of the BM cells suggested alterations in granulopoiesis, which were further confirmed with classical flow cytometry. In detail, BM and peripheral organs of mice engrafted with *TET2*^mut^ human HSPCs were characterized by an increased proportion of neutrophils (CD66b^+^CD15^+^CD16^hi^), which showed low granule complexity. Further analyses revealed an increased number of primary granules and reduced abundance of secondary and tertiary granules in *TET2*^mut^ neutrophils as well as increased levels of myeloperoxidase and neutrophil elastase in these cells. In line with these notions, RNA-Seq analysis showed that *TET2*^mut^ neutrophils show transcriptional profiles characteristic of immature stages and low-density neutrophils, as well as a downregulation of pathways related to phagocytosis, neutrophil activation, and response to infection. Of note, upregulation or downregulation of gene expression did not directly correspond to the methylation status of gene promoters. Further integration of the methylation landscape with assay for transposase-accessible chromatin sequencing data showed that only a subset of gene promoter regions were both hypermethylated and less accessible in the differential accessible peak analysis. These promoter regions were enriched in binding motifs associated with E2F, KLF3, or SP1 TFs, suggesting that these transcriptional regulators, which are involved in terminal neutrophil maturation, are less active in *TET2*^mut^ neutrophils.

Quin *et al*. (96) studied the effects of TET2 deficiency in the hematopoietic system, using a transgenic line expressing CRE recombinase under the control of the *Vav1* promoter to remove exon 3 of the *Tet2* gene. In this model, already under basal conditions, TET2 deficiency led to expansion of monocytes (CD45^+^), a trend for increased blood neutrophil counts, and higher numbers of immature neutrophils (CD11b^+^Ly6G^+^CD101^–^) in circulation, which suggests a maturation defect in these cells. Also in human CHIP carriers, higher numbers of total monocytes, classical monocytes, and neutrophils were documented, although a more detailed analysis of *TET2* mutation carriers was not reported.

Expansion of monocytes and neutrophils caused by TET2 loss does not result in more efficient defense against bacterial infection (96). Strikingly, TET2-deficient mice showed increased pathology, impaired bacterial clearance, and higher mortality upon infection with *Streptococcus pneumoniae*. Of note, although the proportion of circulating neutrophils was higher in TET2-deficient mice than in the control animals, neutrophil infiltration to lungs was decreased in these mice during infection. Further analyses showed that TET2-deficient neutrophils have compromised immune functions reflected by impaired motility, phagocytosis of bacteria, and neutrophil extracellular trap formation. In line with these notions, CHIP carriers expressed lower levels of the high-affinity Fc-γ receptor, CD64, on circulating neutrophils (although the exact mutation in the patients was not reported in this study). Subsequent RNA-Seq analysis showed that murine *Tet2*^−/−^ neutrophils demonstrate a proinflammatory signature and downregulation of the pathways related to motility and migration.

Thus, TET2 dysfunction leads to alterations in immune cell responses, in particular related to expansion and proinflammatory activities of myeloid cells. These observations provide a potential explanation for the clinical consequences of TET2 deficiency described in the following section.

## TET2 deficiency in the diseases associated with inflammation

TET2 deficiency in the immune cell lineage, apart from the mutations leading to leukemias, is mostly studied in the context of CHIP (see the “Alterations of hematopoiesis triggered by TET2 dysfunction" section). TET2-CHIP is characterized by the expansion of clones of immune cells with dysfunctional TET2 and has been linked to a higher risk of numerous age-related diseases with an inflammatory component. TET2-CHIP is characterized by constantly elevated blood levels of IL-1β, IL-6, and IL-8, which resembles chronic inflammation (80). Overall, it is assumed that loss of TET2 in immune cells leads to excessive inflammation, which is an unfavorable factor worsening the pathology. It should be noted, however, that recent studies have challenged this view, and the results of TET2 deficiency might be both detrimental and beneficial, depending on the pathological context.

The pathophysiological relevance of TET2-CHIP has been studied for ischemic stroke, atherosclerosis, cardiac failure, and AD. Animal models used in these studies were designed to recapitulate the features of CHIP by partially replacing the WT BM with TET2-deficient cells, giving rise to mutated clones. These models often rely on transplanting a mixture of WT and TET2-deficient cells to lethally irradiated mice. Such strategies will be referred to as the TET2-CHIP models herein.

### Cardiovascular diseases

**Figure 6:**
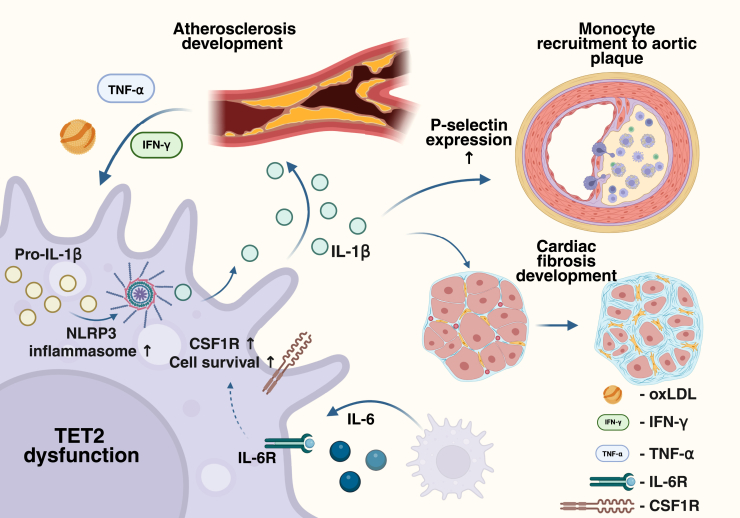
**Involvement of TET2-deficient macrophages in cardiovascular diseases**. TET2-deficient macrophages show higher survival and prolonged proinflammatory activation in the context of inflammation and are recruited at higher rates to the aortic plaque. Please see the main text for the detailed description. TET2, ten–eleven translocation 2.

Probably the best documented association of TET2-CHIP with human disease links it to the risk of cardiovascular diseases, in particular atherosclerosis, coronary heart disease, and myocardial infarction (80, 113, 114, 115, 116). Studies in murine models have shown that mutations in *Tet2* can directly contribute to disease by augmenting the inflammatory activities of leukocytes (Fig. 6) (38, 97, 99, 117).

The causative role of TET2-CHIP in atherosclerosis has been demonstrated in several studies in which the results of TET2 deficiency were examined in atherosclerosis-prone mice deficient for low-density lipoprotein receptor (*Ldlr*^−/−^) fed with high-fat/high-cholesterol diet. In these conditions, TET2 deficiency in myeloid cells has repeatedly been shown to accelerate atherosclerotic pathology, both after a complete BM replacement with TET2-deficient cells (113) and in the TET2-CHIP model (38). In these studies, different strategies were used to achieve TET2 deficiency: deletion of exons 8 to 10 (38) or exon 3 (113), resulting in similar phenotypes. More detailed examination of the pathological phenotypes revealed that TET2-CHIP mice showed enhanced inflammatory profiles of macrophages, in particular increased release of IL-1β (38). In line with this notion, NLRP3 inhibition led to a reduction in plaque size, which was greater in TET2-CHIP mice than in WT animals. A more recent study by Liu *et al*. (99) again confirmed the causative role of TET2-CHIP in atherosclerosis but pointed to an IL-6-dependent mechanism. In the atherosclerosis model, TET2-CHIP mice were characterized by greater lesion area, accompanied by increased monocytosis and aortic infiltration by monocytes and macrophages, as well as by increased CSF1R staining in lesional macrophages. All these phenotypes were reversed by a treatment with an anti-IL6R antibody, demonstrating the causality of IL-6 signaling in TET2-CHIP–associated atherosclerosis.

The role of TET2-CHIP in promoting aberrant tissue remodeling after cardiac dysfunction has been studied in several experimental models. In essence, greater postinfarction heart tissue remodeling and more severe heart dysfunction were seen in TET2-CHIP mice in these studies (97, 117). This phenotype was accompanied by increased expression of several genes (*Il1b*, *Il18*, *Cxcl2*, *Ccl2*, *Ccl5*, and *P-selectin* but not *Il6* and *Tnfa*) in the noninfarcted marginal zone in TET2-CHIP mice in the model of permanent ligation of the left anterior descending artery (97). Furthermore, the number of CD45^+^ immune cells, including macrophages, B cells, and CD3^+^ T cells, was increased in the myocardial tissue from TET2-CHIP mice. Of note, NLRP3 inflammasome inhibition reverted enhanced postinfarction tissue remodeling in these mice, substantiating the causative role of increased IL-1β in the observed phenotype. Similar results were obtained in two other models of cardiac failure: transverse aortic constriction (97) and the angiotensin II infusion model, which drives hypertension leading to cardiac dysfunction (117).

### Brain ischemia

Mutations in *TET2* have been associated with overall stroke risk, and in particular, ischemic stroke, which accounts for approximately 85% of all stroke cases. In detail, meta-analysis of CHIP and stroke risk in the general population (118) showed that CHIP was associated with an increased risk of total stroke after adjustment for age, sex, and race. With respect to gene-specific association results, *TET2* showed the strongest correlation with total and ischemic stroke. Furthermore, CHIP was associated with large artery stroke, recurrent vascular events, and all-cause mortality in first-ever ischemic stroke patients (109). TET2-CHIP was associated with a higher risk of composite end-point occurrence in multivariable analysis, and the serum IL-1β levels in patients with *TET2* mutations were higher than in CHIP-negative patients. It should be noted that ischemic stroke risk and major adverse events evaluated in this study are largely driven by underlying cardiovascular disease, particularly atherosclerosis, which itself is known to be promoted by TET2-CHIP. Interestingly, increased risk of recurrent vascular events and death seen in patients with *TET2*-mutated clones was partially mitigated by a common germline variant of the IL-6 receptor (IL-6R p.D358A), suggesting that the interplay between TET2 deficiency and IL-6 signaling may be an underlying cause of the increased risk seen in TET2-CHIP patients.

The role of TET2-CHIP in ischemic stroke has been recently evaluated in an animal model (119). In the acute phase (24 h) after the ischemic stroke model (middle cerebral artery occlusion), TET2-CHIP (with exons 8–10 removal) had no impact on stroke volume, edema, infiltration of macrophages and neutrophils, cytokine levels, or neurological outcome. However, TET2-CHIP mice showed improved neurological outcomes in the subacute phase (14 days after stroke). Surprisingly, this improvement was accompanied by dampened inflammation, evidenced by reduced astrogliosis and lower transcript levels of multiple proinflammatory cytokines in bulk RNA-Seq of brain tissue samples. These results raise the question regarding the underlying mechanisms that appear to be different than those documented for TET2-dependent gene expression regulation in macrophages studied thus far in various setups mimicking inflammation (Table 2).

### Alzheimer’s disease

Cells bearing CHIP-associated mutations, presumably originating from circulating blood cells, are found in the brains of aged patients (111, 120), supporting the possibility that CHIP cells may have a direct impact on neurodegeneration. However, association studies in humans have brought conflicting results regarding the impact of CHIP on the risk of neurodegenerative diseases, including AD (111, 121). Importantly, the results of these early studies might have been obscured by the fact that they did not distinguish between various mutated genes causing CHIP. More recently, it became clear that while CHIP overall does not significantly alter the likelihood of developing late-onset AD, TET2-CHIP is associated with a lower risk for this disease (111) and this association appears to be unique among other CHIP-related mutations. The potential protection from AD pathology by TET2-CHIP was further evaluated in a model of AD (5xFAD mice overproducing Aβ), additionally challenged with weekly low-dose LPS injections (which will be referred to as AD + LPS). Under these conditions, mice that were subjected to complete BM replacement with TET2-deficient cells showed better memory functions and a reduction in the number of Aβ plaques in the brains compared with AD + LPS mice that received WT BM. Of note, peripheral BM-derived immune cells infiltrated the brain and replaced brain-resident microglia, gaining microglia-like properties as expression of the Tmem119 marker. This phenomenon was enhanced in TET2-CHIP mice. The scRNA sequencing analysis revealed significant upregulation of cytokine response and chemotaxis pathways in infiltrating macrophages in TET2-CHIP mice compared with control AD + LPS mice. This included elevated expression of chemokines and their receptors, such as *Ccr2*, *Ccr1*, *Ccl4*, *Ccl6*, *Cxcl2*, and *Cxcl10*. Moreover, TET2-CHIP mice showed an increase in nonclassical Ly6C^−^ monocytes and M1 inflammatory macrophages in the AD + LPS model. Another study documented an increase of Iba1^+^ microglia, IL-6, TNF-α, and IL-1β in AD model mice after TET2 depletion, corroborating the findings that TET2 deficiency enhances inflammatory response (122). In this work, however, depletion of TET2 led to increased Aβ plaque formation in the hippocampus. It should be noted that the model in this study did not recapitulate the features of CHIP: *Tet2* expression was silenced locally in the brain with the use of an shRNA-based approach.

### TET2-CHIP—perspectives in personalized medicine

Despite *TET2* being one of the most frequently mutated genes in CHIP, studies comparing the effects of *TET2* mutation type (canonical *versus* noncanonical) on the functional properties of immune cells are still missing. Studies in mice documented the differential effects of the *Tet2* catalytic mutation *versus* complete *Tet2* depletion on BM and hematopoiesis; however, their impact on the activities of effector cells like macrophages and the immune response remains to be elucidated (21). The type of *TET2* mutation can have important functional consequences for patients. Pronier *et al*. described elevated levels of MIF in BM and monocytes of patients with truncated TET2, compared with individuals with WT TET2. Of note, patients with a nontruncating *TET2* mutation did not show such alterations (40), pointing to the different implications of the two types of mutations in *TET2* in humans. Truncation of TET2 results in the loss of its ability to bind its interacting partners EGR1 and HDACs, which affects its noncanonical function and the regulation of *Mif* expression. Since the repertoire of TET2 interactors is broad (31, 32) (see “The mechanisms of TET2-dependent regulation of gene expression” section), truncating mutations may also influence the expression of other cytokines.

Further preclinical and early clinical observations suggest that targeting downstream inflammatory pathways (NLRP3/IL-1β, IL-6R, and STING) may mitigate the cardiovascular and hematologic consequences of TET2-related clonal hematopoiesis, although mutation-specific effects have not yet been defined. Individuals with CHIP carrying a common IL6R variant that dampens IL-6 signaling (p.Asp358Ala, rs2228145-C), a genetic proxy of pharmacological IL-6R blockade, have a reduced risk of incident cardiovascular events (123). Consistently, IL-6R antibody treatment reverses TET2-CHIP–related atherosclerosis in mice, supporting IL-6R inhibition as a rational strategy in TET2-CHIP–associated vascular disease (99). In parallel, multiple experimental models show that TET2 deficiency enhances NLRP3 inflammasome activation and IL-1β production and that pharmacological NLRP3 inhibition attenuates heart failure (97), atrial fibrillation, and atherosclerosis phenotypes (38, 124, 125). In agreement with these notions, results from the CANTOS clinical trial indicate that patients with TET2-CHIP present greater benefit from IL-1β blockade with canakinumab than non-CHIP carriers (126). Finally, recent work implicates STING activation in aberrant hematopoiesis and leukemogenesis driven by TET2 mutations. STING inhibitors, such as H-151, selectively suppress colony formation and leukemic expansion of TET2-mutant AML cells in patient-derived xenograft models, suggesting that STING is a druggable target in TET2-driven myeloid neoplasms (89). Nevertheless, given that catalytic and truncating *TET2* mutations yield distinct immune phenotypes, mutation type should be considered in future mechanistic and therapeutic studies. In particular, further studies on the functional implications of various types of mutations in *TET2* for immune cell functions and their clinical consequences are needed. The conclusions of such studies are crucial to evaluate the potential of patient stratification by mutation type in future treatments of TET2-CHIP–related disorders.

## Conflict of interest

The authors declare that they have no conflicts of interest with the contents of this article.
